# The University and Hospital Bulletin

**Published:** 1905-06

**Authors:** 


### The University and Hospital Bulletin

DEVOTED TO THE INTERESTS OF THE UNIVERSITY OF BUFFALO AND THE HOSPITALS.

EDITED BY
NELSON W. WILSON, M. D.

*Assistants:*
E. R. McGuire, M. D., Burton T. Simpson, M. D.

Under the auspices of the Faculty of the Medical Department of the University

M. D. Mann, A. M., M. D., Dean,
Charles G. Stockton, M. D.,
John Parmenter, M. D.,
Eli H. Long, M. D.,
Roswell Park, A. M., M. D., LL. D.,
Charles Cary, M. D.,
Herbert M. Hill, A. M., Ph. D.,
Herbert U. Williams, M. D.

AND

*Buffalo General Hospital* Henry Reed Hopkins, M. D.
*Hospital of the Sisters of Charity* Eugene A. Smith, M. D.
*German Hospital* Herman E. Hayd, M. D.

### University of Buffalo—Commencement Week, '05.

THE exercises pertaining to the fifty-ninth annual commencement of the department of medicine, the eighteenth annual commencement of the department of pharmacy and the thirteenth annual commencement of the department of dentistry were held at the Teck theater, Thursday morning, May 4, 1905, at 11 o'clock.

### MEETING OF THE COUNCIL.

In the early part of the day the annual meeting of the council of the university was held. The vice-chancellor, Charles P. Norton, presided, and the recommendation of the several faculties relating to the candidates for the degrees in medicine, pharmacy and dentistry was confirmed. During the year the Hon. George Gorham resigned the office of vice-chancellor and Mr. Charles P. Norton, a prominent member of the Buffalo Bar, was elected to fill the vacancy.

### ALUMNI ASSOCIATION.

This association departed from its usual custom and held its meeting this year during two days. An elaborate program was prepared by the executive committee which was observed with strict adhesion to detail. The scheme included a series of clinics at the several hospitals and at the university, an executive and scientific meeting on Wednesday evening, May 3, at alumni hall, commencement exercises at the Teck theater on Thursday, luncheons at the several clubs, and the whole concluding with a banquet Thursday evening at the Iroquois hotel. The following is a detail of the program:

*Wednesday, May 3, 1905.*

BUFFALO GENERAL HOSPITAL.

10 a. m.—Cerebrospinal meningitis, DeWitt H. Sherman.

10.30 a. m.—Persistent branchial cleft, Irving M. Snow.

10.30 a. m.—(*a*) Operation for prolapsed iris; (*b*) Presentation cases, Lucien Howe.

11 a. m.—(*a*) Sequelæ of pneumonia; (*b*) Endocarditis, DeLancey Rochester.

11 a. m.—(*a*) Ventral hernia; (*b*) Tubercular joint, William C. Phelps.

12 m.—Myelitis, James W. Putnam.

ALUMNI HALL—UNIVERSITY—LECTURE HALL.

10.30 a. m.—Tubercular knee, Bernard Bartow.

11 a. m.—(*a*) Gastric cancer following ulcer; (*b*) Perforating gastric ulcer, A. L. Benedict.

11.30 a. m.—(*a*) Renal decapsulation; (*b*) Colecystenterostomy, Eugene A. Smith and Charles Sherman Jewett.

12 m.—(*a*) Tracheotomy; (*b*) Cystic goitre; (*c*) Radical ear operation with Brain sinus, George F. Cott.

12 m.—Gunshot wound of head, A. W. Bayliss.

UNIVERSITY CLUB.

1 p. m.—Luncheon, resident alumni to out-of-town alumni. Cards to be obtained of executive committee.

HOSPITAL SISTERS OF CHARITY.

3 p. m.—(*a*) General peritonitis; (*b*) Nephropexy; (*c*) Perforating ulcer of foot; (*d*) Presentation of cases, Edward J. Meyer.

4.30 p. m.—(*a*) Spleenic anemia; (*b*) Arthritis deformans; (*c*) Endocarditis, Henry C. Buswell.

ALUMNI HALL—COLLEGE BUILDING.

8 p. m.—(*a*) Reading minutes previous meeting; (*b*) appointment of nominating committee; (*c*) report treasurer; (*d*) report trustees; (*e*) report executive committee; (*f*) miscellaneous business; president's address, DeLancey Rochester; address, E. L. Shurley, Detroit, Mich.; (*g*) election officers.

LIBRARY—COLLEGE BUILDING.

11 p. m.—Smoker, by invitation of the faculty.

*Thursday, May 4, 1905.*

INVITATION CLINICS.

9 a. m.—Woman's Hospital—C. C. Frederick—Abdominal sections.

10 a. m.—University—Alumni Hall—E. and G. W. Wende—Mycosis fungoides.

10.30 a. m.—German Hospital—Julius Ullman—(*a*) Typhoid; (*b*) Multiple neuritis; (*c*) Pseudoleukemia; (*d*) Articular rheumatism.

10.30 a. m.—University—Alumni Hall—T. H. McKee—Nerve suture.

11 a. m.—Sisters of Charity Hospital—L. G. Hanley—Nephropexy.

TECK THEATER.

11 a. m.—Commencement exercises.

SATURN CLUB.

1 p. m.—Luncheon to association, by Drs. Mann, Park, Stockton, Cary, Snow, Williams and Rochester.

BUFFALO GENERAL HOSPITAL.

3 p. m.—Surgical clinic, Matthew D. Mann.

3 p. m.—Medical clinic, Charles G. Stockton.

4.30 p. m.—Surgical clinic, Roswell Park.

4.30 p. m.—Medical clinic, Charles Cary.

HOTEL IROQUOIS.

7 p. m.—Annual banquet.

### COMMENCEMENT CEREMONIES AT THE TECK.

Some time before the appointed hour every available seat in the theater was occupied by friends of the college and graduates who had assembled to witness the ceremonies and to greet the candidates for degrees. At 11 o'clock the classes of medicine, pharmacy and dentistry, numbering in the aggregate about 150, marched to their appointed seats immediately behind the orchestra, during which appropriate musical selections were played. Next came the faculties, approaching from the rear of the stage, led by the vice-chancellor, supporting the Rev. Richard Earle Locke, who took seats upon the stage.

### DEPARTMENT OF MEDICINE.

The graduating ceremonies began with prayer by Rev. Frank Hyatt Smith, which was followed by a musical selection and then the class in medicine, numbering thirty-seven, was presented to the vice-chancellor by Professor Eli H. Long, M.D., secretary of the faculty; meanwhile, the dean, Professor Matthew D. Mann, M.D., administered the Hippocratic oath, after which the degree of doctor in medicine was conferred by the vice-chancellor, Charles P. Norton, Esq., upon the following named successful candidates:

Andrews, Herman David, Buffalo
Beach, Channing Elias, Buffalo
Becker, George Adam, Buffalo
Braner, Harry Edward, Buffalo
Burlingham, William Bernard, Buffalo
Bethune, Charles Williams, Buffalo
Cannon, Hadley Thomas, Elmira
Cohn, David, Buffalo
Connors, Thomas William, Buffalo
Eames, Lewis Nelson, Lee Center
Fiero, Carl M., Peoria

Fisk, George Clayton, Belfast
Flannery, John Matthew, Buffalo
Foster, Edwin Carlton, Hammondsport
Good, Norton Henry, Buffalo
Hengerer, Louis, Buffalo
Hill, Stephen Mortimer, Cazenovia
Jackson, George Breton, Caneadea
Kavinoky, Samuel, Buffalo
Johnson, Herman Walter, Gowanda
Lande, Abraham, Elmira
Lemen, Fred'k Michael, Dansville
Linklater, Eugene Ross, Buffalo
McKenney, Descum Clayton, Buffalo
Mott, Albert Elgin, Bowmansville
Padelford, Charles Eugene, Padelford
Pchellas, Victor Albert, Buffalo
Peaslee, Joseph Asher, Randolph
Perkins, Frank Elliott, Copenhagen
Prudden, William Hopkins, Lockport
Reimann, Edmund Philip, Buffalo
Schaefer, Arthur Charles, Buffalo
Sernoffsky, Isaac, Buffalo
Schweitzer, Joseph, Buffalo
Simpson, Leo Francis, Rochester
Sperans, Joel, Buffalo
Sullivan, William Joseph, Dunkirk

The dean of the faculty, Professor Mann, then announced the honor roll, including the standing of each member, as follows:

| | |
|---|---|
| Lewis Nelson Eames, Lee Center, N. Y. | 95 % |
| Leo Francis Simpson, Rochester, N. Y. | 92.88 " |
| Albert Elgin Mott, Bowmansville, N. Y. | 91.51 " |
| Stephen Mortimer Hill, Cazenovia, N. Y. | 91.40 " |

### DEPARTMENT OF PHARMACY.

The candidates for the degree of bachelor in pharmacy, thirty-six in number, were presented by Professor John R. Gray, M.D., secretary of the faculty of the college of pharmacy, and the vice-chancellor conferred degrees upon the following named candidates:

*Roy Vincent Agrelius
William Adsit Bryant
*John Buettner
Joseph Henry Callahan
Joseph T. Wesley Coble
Wilber Ray Davis
Arthur G. Drake
Bert Henry Gifford
James Bernard Harrington
Max Himmelfarb
George Daniel Hull
*William Dikeman Hulse
*Charles William Janke
Mary Evangeline Kelly
M. Frank H. Kenny
Ernest Lambert
John Leffler
Edgar Howard Lincoln
Charles Theodore Mann
Hubbard J. Meyers
*Benjamin Francis Miles
Gates Markham Minckler
William George Overocker
Eugene Ashley Putney
*Walter Scott Redfield
Harold F. Rising
William Andrew Robison
Edward P. Ryan
Frank Whitney Shaw
J. Lee Sherlock
Edward William Shinners
Howard A. Stover
Otto Ernest Tannhauser
Thomas Elba Tefft
Theodore Floyd Young
Elmer E. Zacher

The degree of Master in Pharmacy was conferred upon *Homer E. Dyke.

Professor Willis G. Gregory, M.D., dean of the faculty, announced that Roy Vincent Agrelius, an honor graduate of this

* Diploma withheld on account of being under age.

department, had won the W. H. Peabody prize of $50; and, further, that the honor roll consisted of Roy Vincent Agrelius, William Dikeman Hulse, Edgar Howard Lincoln and William George Overocker.

DEPARTMENT OF DENTISTRY.

The candidates for the degree of doctor in dental surgery, numbering seventy-four, then were presented by Dr. George B. Snow, dean of the faculty, and the vice-chancellor conferred degrees upon the following members of the class:

Albert Edwin Atkinson
Heman Wing Backus
Roscoe Lyman Barber, B.S.
*Frank Albert Beyer
Irving M. Billington
Frederick Edwin Bryant
Charles Frank Bullock, A.B.
Cephas Rowland Campbell
Daniel D. Carmichael
Walter Frank Chappelle
Clark George Cole
Hugh Cunningham
Burt Brook Davis
Albert Francis Dyer
Harry Franklin Eckler
Seward Crapser Edgerly
Frank Washington Edmunds
George M. A. Empey
James Edward Espie
Frank Matthew Evans
Ernest John Eveleigh
Guy Maxwell Fiero
Allen Joseph Fraley
Edgar Sumner Gill
Carl DuMelt Gurnee
Frank James Handy
Dan Raymond Howe
Charles Hugh Irish
Joseph Justus Jacobson
Frederick Henry Jelley
Harold Francis Jones
*George Burnett Kehr, Ph.G.
Milton Wesley Kohler
Frank Huntington Kulp
Clarence Thomas Lansing
Fred Pierson Leigh
Alma Van Peyma Lloyd
John Luke Maxwell
*Floyd Eugene Metcalf
Charlie Orland Middleton
Clarence George Morsheimer
Margaret Anna Munroe
William Arthur Myers
Everett Ray Neff
Ferris David North
Harry Warford Ogden
Charles Andrew Pankow
Roy Clinton Parker
John Harmon Parmele
Foster Samuel Post
Roy Wilbur Reid
Frank Augustus Rheubottom
Frank Elmer Rians
J. G. Roberts
Roy George Roberts
Walter Mark Rooks
Alexander Ross
*Adolphus Augustus Rounds
Oscar Garfield Ryerse
Frederick James Shaddock
Orin Merrill Skinner
William Alexander Smith
Charles Conrad Steigerwald
*Amos George Stiker
George Albert St. John
Laverne Coleman Swain
Walter Edward Thomas
*Clarence Thorn Van Woert
Clarence Adna Warner
Walter Ambrose Warren
James Edward Watson
*Harold Parker Wells
Charles William Wise
William Elphiston Wray

The dental graduates who received honors are Heman Wing Backus, Frederick Edwin Bryant, Clark George Cole, Albert Francis Dyer, Frank Washington Edmunds, Carl DuMelt Gurnee, Oscar Garfield Ryerse, Orin Merrill Skinner, Amos George Stiker and Harold Parker Wells.

* Diploma withheld on account of deficiency in time.

A musical selection was then rendered during which the ushers occupied themselves with the distribution of flowers to the graduates. These had been sent in profusion by their friends and stacked upon the stage front, as well as in the area behind the stage. This was a feature of great interest and added variety to the occasion. The color and fragrance of the flowers gave a pleasant suggestion of spring and served to bring the audience as well as the recipients of the trophies in closer touch with nature. Formerly, the faculty ruled against the presentation of flowers at commencement exercises, but we are glad to see the custom revived. It lends attractiveness to the scene and is befitting the occasion.

The address to the graduated classes was delivered by the Rev. Richard Earle Locke, pastor of Calvary Presbyterian church, Buffalo, and will be published hereafter. It was an address full of human nature and one which appealed to the manliness of the audience, whether graduates or spectators. A benediction and music served as a finale to a most appropriate observance of commencement ceremonies.

The alumni present immediately gathered at the Saturn Club, where they were entertained at luncheon by Drs. Matthew D. Mann, Roswell Park, Charles G. Stockton, Charles Cary, Irving M. Snow, Herbert U. Williams and DeLancey Rochester. Just as the luncheon was concluding, Dr. A. L. Benedict, of Buffalo, proposed a vote of sympathy to Professor Charles G. Stockton in the grievous affliction which had befallen him. Dr. Benedict was instructed to communicate in writing to Dr. Stockton the action of the association.

### THE BANQUET.

As an appropriate conclusion to the commencement ceremonies and alumni association proceedings a banquet was served at the Hotel Iroquois, Thursday evening, May 4, at 7 o'clock. At this function were seated the faculty, the alumni and the new graduates and guests, numbering more than 100 in all. Dr. DeLancey Rochester, president of the alumni association, presided at the feast and acted as toastmaster—a dual position in which he served with élan. The banqueters were entertained between courses and speeches by the University quartette: Fred Hicks, first tenor; Edward Cox, second tenor; George McIntire, first bass; and Dr. W. L. Goodale, second bass.

When coffee and cigars were served the toastmaster began the introduction of the speakers. He spoke of the alumni association, the work that it had performed in the past and in particular of the present meeting now nearly concluded. In the course of his remarks he referred to the laborious duties of the executive committee and called upon the chairman, Dr. Albert T. Lytle, to speak to the first toast,

"THE ALUMNI ASSOCIATION."

Dr. Lytle in his response called attention to the fact at this the thirtieth anniversary dinner, that though there were over 2,000 graduates of the medical department there were less than 500 active members of the association; that efforts had been made to maintain interest in the association and in the medical department by offering each year some entertainment in character both serious and otherwise, but owing to the poor response on the part of the individual graduates to pleas for aid by becoming active members of the association it had always been difficult to attract members from a distance to attend the annual meetings. Like the sinews of war, money is an essential factor in the successful operation of an alumni reunion; the faculty of the medical department, however, has ever been generous in aiding the association to keep in touch with the alumni and to make the social events of each reunion a success.

After several years of experiment it was thought best to offer to the alumni at this meeting something like a post-graduate course, availing ourselves of the advantages of the vast amount of clinical material under the care of members of the association resident in Buffalo. When one realises that every eleemosynary institution in Buffalo has several alumni upon its staff, each of whom is a capable instructor, it is not surprising that it should be considered feasible to make such a course interesting and instructive. The executive committee believes that with the generous aid of these gentlemen a clinical course of six days could be given to the graduates of the medical department, not only in general medicine and surgery, but in all the important specialties. Such a course would secure for each alumnus attending, instruction in one week equal to that of four or five in any post-graduate school of instruction elsewhere, and all for the value of an active membership in our association.

The experiment made this year proves how much material there is at hand for use for the benefit of members of the association, hence it is proposed that the clinics next year shall be extended over a much greater period of time, so that there will be opportunity for more variety and less conflict in hours. The executive committee of the association desires to acknowledge its obligation to each and every one who so willingly and kindly offered his services for the entertainment and instruction of the members. Dr. Lytle interspersed his speech with a number of amusing anecdotes which provoked laughter and contributed to the enjoyment of the occasion.

The toastmaster then made reference to the fact that an attempt is making to establish a college of arts, and said that the university was receiving on all hands much encouragement, indicating that the movement would meet with success if properly handled. In view of this he had asked the vice-chancellor, Charles P. Norton, Esq., to respond to the next toast,

"THE UNIVERSITY."

Vice-chancellor Norton began his remarks by saying that he wanted to speak to the medical department about the work that was before the university to which Dr. Rochester has just alluded. He thought that the traditions of the university should appeal especially to the medical department because, until the department of pharmacy was created in 1886, the medical department was all there was of the university, and so should be instinct with the traditions of its founders. He remembered as a little boy, many of these men at his father's house and their talk of the future of the city. They foresaw a great future for Buffalo, and as one of its fundamental institutions obtained from the legislature the charter of this university. This was when the city was but a dozen years old and had a population of only 30,000 people. But what the pioneers and founders of the university had foreseen, has now come to pass. Buffalo has now become a city of 400,000 people. It is the eighth in order of population of the cities of the country. It will soon be one of the great cities of the world. And the time has now come in the history of the university to round out the scheme of its founders, and to create other departments for the university, as Austin Flint, Frank Hastings Hamilton, James P. White, and Thomas F. Rochester have already developed the medical department.

A college education is now necessary to a well educated man or woman to compete successfully for the positions of life. Buffalo is the only city of its size in the country that has not already recognised this fact and which does not have a college for its citizens within its limits or within ten miles of its boundaries. The first work to be done is to awaken the citizens of Buffalo to the real need of such a university, and also to awaken them to the fact that the educational standards and requirements of the present day have advanced far beyond those even of the last generation. Fifty years ago the district and the parochial school gave to the community all the education that was demanded. Until quite recently, the public, the parochial, and the high schools have fully equipped young men or young women for the ordinary positions in life. But at present and for the future more will be required. College men and women are becoming more and more common every day. Soon to be a college man or woman will be the rule and not to be a college man or woman, the exception. They are now wanted as superintendents of shops, on newspapers, in the professions, and as heads of departments in various kinds of business, and unless they are college men or women, the children of the poor cannot be expected to rise to those positions. The educators understand this.

At a speech delivered at the conference of the delegates of the various alumni associations of Buffalo, Mr. Emerson, superintendent of education, said, if a school of letters was founded in Buffalo, within five years it would have five hundred students. Mr. Detmers, principal of the Lafayette high school, said that of the young men and women, graduates of the high schools of the city of Buffalo, each year, there were many whose talents richly deserved the higher education, but who cannot afford to leave town to go to college; and thus the community is deprived of the benefit of this talent.

The object of the movement is to supply this want and to create a higher school for the benefit of the citizens of Buffalo. The movement aims to enlist the sympathies, and interest, and pride, and patriotism of every citizen of Buffalo, so that the people of Buffalo may feel that the college is their own.

The medical department has already taken an active part in this movement. Dr. Mann has done much work. Dr. Park, on April 15, made a speech to the Harvard Alumni Association, which led to the appointment of a committee to consider the ways and means of creating a college of liberal arts for the University of Buffalo. The resolution also called upon the alumni of the other universities to coöperate with the Harvard alumni. Nineteen colleges have responded. On April 27 a conference of these colleges was had and an executive committee appointed, so that it may now be said that the university men of Buffalo are organised, ready, and waiting to carry on the movement.

The vice-chancellor concluded by urging upon the medical department the duty of taking an active hand in this movement. He said that he did not think that any body of men in the community had so great private influence as physicians, and he implored them to exert it in the direction named. Buffalo, he said, in fifty years, would be a city with a very large population and would be the metropolitan city of a neighborhood with a population numbering in the millions. A college would certainly come. But now was the time to create an intellectual center for Buffalo, so that as the town increased in size, controlling influence would be exerted by a well organised university, which had been created by the citizens themselves and in which the people who erected it would take pride.

The speech of Mr. Norton was delivered with great fervency, and its eloquence was appreciated by the banqueters who frequently interrupted its delivery with applause.

The toastmaster then announced that the Right Reverend Charles H. Colton, D.D., Bishop of Buffalo, would respond to

"THE CLERGY."

Bishop Colton, with characteristic simplicity of manner and speech, made a touching allusion to the relationship of the clerical and medical professions; to their mutual interests in working for the benefit of humanity; and, especially, to their interdependence in ministrations to the sick and dying. He recounted his pleasant relations to physicians which had existed ever since he entered the priesthood, and which he predicted would continue until the end of his spiritual administration. He spoke in the highest terms of the self-sacrificing nature of the physician's calling and recited parallels in that of the clergy; in particular, he called attention to the dangers often entailed by the faithful performance of the duties of each profession,—dangers which deterred neither physician nor priest from rendering loyal service to the afflicted.

Bishop Colton's address was full of christian spirit and benevolent precept,—commanding attention from beginning to end and eliciting the generous plaudits of his hearers during its delivery.

To the next sentiment Dr. Rochester said much thought had been given because of its importance, and that after mature deliberation Dr. Marshall Clinton had been invited to respond to

"THE LADIES."

Dr. Clinton said that to appropriately present, on an occasion like this, the many charms and blessings of the fairer sex he found quite beyond his powers. To toast and laud the part they have taken in our profession, he continued, is more feasible. My first experience, he said, with the gentler sex in collegiate work began some thirteen years ago when a lone girl entered the budding medical class of 1895 at Niagara University. The disgusted amazement of many of the class at her effrontery in daring to intrude an influence that stood for something better than the utterly flippant and the bizarre was beyond description. Much murmuring was heard—and disagreeable remarks were passed to test the feeling current with the majority of her classmates. That innate chivalry which lies like a sentient watchdog at the gateway of our better acts, sprang up and organised a group of men who served notice on the class at large that any molestation with that girl student would be followed by personal chastisement.

I doubt if that girl in her quiet hardworking life ever knew how much her influence stood for in the whole school. At best our profession is what might politely be considered an unconventional study, and to our girl students we owe the banishment of ribaldry and jest that in other days were considered a part of the everyday duties. We must admit that women have succeeded in our profession when judged by standards of material progress. That they help their patients we know and sometimes we hear of instances in which they vie or even excel the sterner sex in their skill and success.

In the laboratories today some of our best workers and investigators are women. In hospitals they number among the most skilful. In conclusion, let me ask you to join me in a toast to "Our Ladies," and I ask the women of our profession to keep for themselves always,—added to their M.D.'s,—the title of gentlewomen.

The counterpart of the last topic would require great skill to deal with, said the chairman, but fortunately an alumnus equal to the occasion had been found. He introduced Dr. M. Elizabeth Schugens who, in the following fashion, served up

"THE MEN."

It was with no small amount of trepidation that I accepted the signal honor of responding to a toast, the ability to do justice to which might have been a matter of doubt in the mind of a much more confident woman. The subject, "The men" or better "The gentlemen," is so large, so splendid that I cannot hope to give it full credit. A toast to "The men" must, indeed, be spelled out by the word, achievement; for it would be a history of glorious deeds, of chivalry, of service to the world, of noble accomplishments in the domains of the mental, moral and physical,—a history of conquest over self, the development of the home and family life and, what is dearer to us, the placing of woman upon the seat of honor, in that home.

And so we all recognise:

"Two heads in council, two beside the hearth,
Two in the tangled business of the world,
Two in the liberal offices of life,
Two plummets dropt for one to sound the abyss
Of science, and the secrets of the mind."

---

"The woman's cause is man's: they rise or sink
Together, dwarf'd or godlike, bond or free:
For she that out of Lethe scales with man
The shining steps of Nature, shares with man
His nights, his days, moves with him to one goal."

Just as in the perfectly balanced orchestra we have the instruments which give us vigor, martial beauty, strength, frenzy, so must we have those which interpret gaiety, pathos, tenderness, eloquence, sweetness, in order that we may have the whole beauty of orchestral effect,—the symphony.

There is ample work for both, and a definite field in which each may accomplish that work. Man cannot do woman's work nor should woman wish to do man's. The life and happiness of the one are so indissolubly linked with those of the other, that any consideration of man without woman, were impossible. Tennyson has expressed this beautifully, when he says:

"Let her make herself her own
To give or keep, to live and learn and be
All that harms not distinctive womanhood.

For woman is not undevelopt man,
But diverse: could we make her as the man,
Sweet Love were slain: his dearest bond is this,
Not like to like, but like in difference.
Yet in the long years liker must they grow;
The man be more of woman, she of man;
He gain in sweetness and in moral height,
Nor lose the wrestling thews that throw the world;
She mental breadth, nor fail in childward care,
Nor lose the childlike in the larger mind;
Till at the last, she set herself to man,
Like perfect music unto noble words;
And so these twain upon the skirts of Time,
Sit side by side, full-summ'd in all their powers,
Dispensing harvest, sowing the To-be,
Self-reverent each, and reverencing each,
Distinct in individualities."

Surely it was a realisation of this which led this enterprising and progressive university early to open its doors to the women of the community as well as the men. And I add, with pardonable pride I trust, that it was a very wise measure as is amply proven by the excellent work of your colleagues, the women, now in the active practice of medicine.

"Here might they learn whatever men were taught.
Let them not fear: some said their heads were less;
Some men's were small, not they the least of men;
For often fineness compensated size:
Besides the brain was like the hand, and grew
With using;"

And now it seems as if the girls of this city were to share equally in additional benefits, in the establishment of a department of liberal arts in this university. Vice-chancellor Norton has called your attention to the fact that a vigorous effort is being made, not only by the University of Buffalo in all its departments, but also by the alumni of all the universities represented in this city. Will this not afford many girls and boys excellent opportunities for a higher education which they would otherwise be obliged to forego?

And you young men just entering upon your life work as physicians, be not unmindful of the noble men under whose instruction you have sat. They have given you the best things of a lifetime,—all the accumulated knowledge and experience at their command. It is just as true today as it ever was, that a prophet is not without honor save in his own country. Many of your professors enjoy more than local and national fame; their names even are mentioned with honor and respect across the seas.

In closing, permit me to turn your thoughts to one, to whose gentle influence, more than to any other, man owes much of his real strength, tenderness, courtesy,—those qualities which give him the grand old name of gentleman. You will remember that not so many years ago, upon a very important occasion in the city of Washington, all the affairs of state paused for a moment, when a president, an American gentleman, turned to kiss his aged mother.

"Alone, from earlier than I know,
I loved the woman:
Yet was there one thro' whom I loved her, one
Not learned, save in gracious household ways,
Not perfect, nay, but full of tender wants,
No Angel, but a dearer being, all dipt
In Angel instincts, breathing Paradise,
Interpreter between the Gods and men,
Who look'd all native to her place, and yet
On tiptoe seem'd to touch upon a sphere
Too gross to tread, and all male minds perforce
Sway'd to her from their orbits as they moved,
And girdled her with music. Happy he
With such a mother! faith in womankind
Beats with his blood, and trust in all things high
Comes easy to him, and tho' he trip and fall
He shall not blind his soul with clay."

The graduates of 1905 being present in a body, the toastmaster complimented the young physicians upon the success that had attended their struggles for the past four years, as attested by the diplomas they now held, and asked Dr. Leo F. Simpson to respond for

"THE GRADUATING CLASS."

Dr. Simpson, in a few well-chosen sentences, expressed the appreciation by the class of 1905, of the privileges enjoyed in the university during the undergraduate courses, and for the courtesies received at the hands of the faculty and corps of instructors. He also thanked the officers of the alumni association for the invitation to the banquet, and concluded by pledging for himself and his classmates continued loyalty to Alma Mater.

Dr. Henry Reed Hopkins spoke as follows to the

"CENSORSHIP OF THE PROFESSION."

The situation is most delicate and the speaker craves your most considerate and generous indulgence. This is commencement day, the supreme day in many of our lives; we are assembled in banquet hall, and we have dined most royally; the very air is electric and tingling with the attendance of distinguished company, with music, with flowers, with the gracious presence of beautiful women; we have been charmed with words of cheer, inspired by words of singular earnestness and eloquence from our vice-chancellor, and our souls have been strengthened by the fervent utterances of the Bishop beloved. In such an atmosphere and in such environment, I am asked to speak to you upon the censorship of the profession. Surely, contrast was never more striking,—the word and the occasion never more lacking in harmony.

Nevertheless, your committee has taken the responsibility, and I must remind you that in spite of the flowers, the music and the feasting of tonight, there will come the tomorrow, and tomorrow will bring to us the plain things of life,—life not as it should be, not as it might be, but life as it is, and in the real tomorrow there is the need now and then of censorship. Let us make pause for a moment to ask why is it that in practical medical life there must now and then be invoked the authority of law to restrain individuals from the exercise of their individual purposes? Why is it that in the medical profession,—the most learned, the most humane, the most beneficent, the most glorious of all the professions, that there must needs be censorship? The answer though not complimentary to our humanity is easy, the reason is obvious. As it has ever been in all time, so as long as men are human it ever will be,—the army of conquest or of defense will have its camp followers; the bank vault stored with bullion will attract the cracksman; the medical profession with its unrivaled opportunities for confidences and for ministrations will have its fakirs. The camp followers, the cracksmen, the fakirs make it expedient that there should be censors and censorship.

Your speaker has had many years experience as censor, but he has never yet been able to make censorship what would be most desirable,—that is to say, to make censorship at once efficient and popular. I am therefore obliged to make the confession, possibly as news to some of my hearers, that in censorship efficiency is more to be desired than popularity and that a considerable degree of efficiency presupposes that some person has been inconvenienced disagreeably and is likely to find censorship unpopular. Nevertheless, there must be censorship.

These elemental principles of human action and necessity for government were firmly established in the minds of the makers of our state, and accordingly we find early provision made by statute for censorship of the medical profession. Since 1806 this censorship has been in the care and keeping of the state and county medical societies, and that these societies have exercised their great responsibility with a reasonable degree of wisdom is proven by the fact that during the entire century more and more of responsibility, more and more occasions for censorship, have been required of the medical societies by the statutes of the sovereign people.

I am conscious that historical accuracy is to be expected even at the hands of one speaking after dinner, hence do not assume to pose before you, or attempt to maintain that all the important medical legislation of the century has been suggested or even supported by the dominant medical profession of our state. Such is not the fact, for on two important epochs in the medical history of the state our part of the profession has differed distinctly and radically from the legislature, and two important statutes have been enacted against our spirited and even acrimonious opposition. I refer to the statutes of 1857 and of 1865, the former authorising the formation of homeopathic medical societies, state and county; and the latter giving like authority to the eclectic physicians to form medical societies. These societies, homeopathic and eclectic, were to be like in all respects to those authorised by the medical laws of 1806 and 1813.

It will not be a waste of time to spend a moment in reviewing the contests of 1857 and of 1865, that we may read the lessons of those earlier days. The light of history may possibly enable us to meet the more intelligently and the more successfully our present problems. You will therefore please recall that the contest of 1857 was essentially a quarrel between doctors and the cause of that quarrel was a question of therapeutics, a question as to the proper dose of a given remedy, the physicians, who subsequently became known as homeopathic physicians, holding the belief and claiming the right to use remedies in smaller doses than was then the custom. The majority, the dominant party, maintained that such notions of dosing were wrong, were unscientific, and that any man holding such beliefs must be either a fool or a knave. The contention had been in progress since the first publication by Hahnemann of his Organon, in 1810, and was stirred into greater activity by his death in 1844.

The acute stage of the contest was reached when physicians were expelled from county societies by reason of their homeopathic faith and practice, whereupon the aid of the state was invoked. Our side of the contest, the dominant party, were right in their hearts, right in their loyalty to the principles of our art; but wrong in their intolerance, wrong in their estimation of what is freedom in science; and in the contest the minority, the weaker party, the party holding the wrong view of therapeutics, but holding the right view of individual liberty won; and the state decreed that in medicine men should be free to follow the dictates of their own judgment, even if that judgment was opposed to the opinion of the majority.

Again, in 1865, there culminated a similar contest between physicians and again over a question of therapeutics, the minority holding extreme views, as was their right and privilege, as to the value of remedies found in the vegetable kingdom, and the majority holding that such views were unscientific and unwise. Again the state was invoked and as before the state again decided that in medicine men should be free and the physicians who then became eclectic physicians were given the privileges of forming societies, state and county, with the attending power of censorship. In reviewing this bit of the medical history of our state men frequently say that at such and such a time our state recognised the practice of homeopathy and of eclectic medicine. I think it a better judgment of history to affirm that in 1857 and again in 1865 the state of New York decreed that liberty of opinion was of the utmost importance for scientific progress, and that in science, in art, in medicine the individual should have liberty.

Three times within the last decade the dominant part of our profession has had opportunity to repeat the mistakes of the fathers of 1857 and of 1865, and three times we have been wise enough to avoid that fatal blunder; and by a wise opposition we have won the contest. Once we have opposed the Christian scientists and twice we have opposed the osteopaths and our opposition has been upon the same line in each case. We have held, and the state has held with us, that we did not care how a man should practise medicine, but that it was above all else expedient that before a man should undertake to practise medicine he should be tested by the state as to his age, moral character, preliminary education and his knowledge of the sciences of anatomy, physiology, pathology and the like. This plain proposition the legislators can comprehend and to this practical proposition the state gives its support. The refinement of choice whether a given case shall have medical treatment, homeopathic treatment, eclectic treatment, Christian science treatment, or osteopathic treatment is not a question for the layman, the legislator; but is and must ever remain a question for the physician, and that physician is likely to make the wiser choice who has the better knowledge of the art and the science of medicine.

You will remember that since 1806 the censorship of the medical profession has been in the hands of that profession. From the beginning county societies were given authority, first to regulate their own affairs relative to the admission and to the expulsion of members, and in later years the county societies have been given the added authority to see to it that the medical law of the state is duly enforced. The scope of that authority you will have clearly in your minds if you listen to a few lines from the public health law:

> No person shall practise medicine after September 1, 1891, unless previously registered and legally authorised or unless licensed by the Regents and registered as required by this article; nor shall any person practise medicine who has ever been convicted of a felony by any court, or whose authority to practise is suspended or revoked by the Regents on recommendation of a state board. . . .
>
> The Regents shall admit to examination any candidate who pays a fee of $25 and submits satisfactory evidence, verified by oath, if required, that he (1) is more than 21 years of age; (2) is of good moral character; (3) has the general education required preliminary to receiving the degree of bachelor or doctor of medicine in this state; (4) has studied medicine not less than four full school years of at least nine months each, including four satisfactory courses of at least six months each, in four different calendar years in a medical school registered as maintaining at the time a satisfactory standard. . . . (5) has either received the degree of bachelor or doctor of medicine from some registered medical school, or a diploma or license conferring full right to practise medicine in some foreign country. . . . Any person who, not then being lawfully authorised to practise medicine within this state and so registered according to law, shall practise medicine in this state without lawful registration or in violation of any provisions of this article; and any person who shall buy, sell or fraudulently obtain any medical diploma, license, record, or registration, or who shall aid or abet such buying, selling or fraudulently obtaining, or who shall practise medicine under cover of any medical diploma, license, record, or registration illegally obtained, or signed, or issued unlawfully or under fraudulent representations or mistake of fact in a material regard, or who after conviction of a felony, shall attempt to practise medicine, or shall so practise, and any person who shall append the letters M. D. to his or her name, or shall assume or advertise the title of doctor (or any title which shall show or tend to show that the person assuming or advertising the same is a practitioner of any of the branches of medicine) in such manner as to convey the impression that he or she is a legal practitioner of medicine or any of its branches, without having legally received the medical degree, or without having received a license which constituted at the time an authority to practise medicine under the laws of this state then in force, shall be guilty of a misdemeanor, and on conviction thereof shall be punished by a fine of not more than $250 or imprisonment for six months for the first offense, and on conviction of any subsequent offense, by a fine of not more than $500 or imprisonment for not less than one year, or by both fine and imprisonment. Any person who shall practise medicine under a false or assumed name, or who shall falsely personate another practitioner of a like or a different name, shall be guilty of a felony. When any prosecution under this article is made on the complaint of any incorporated medical society of the state or any county medical society of such county entitled to representation in a state society, the fines when collected, shall be paid to the society making the complaint, and any excess of the amount of fines so paid over the expense so incurred by the said society in enforcing the medical laws of this state, shall be paid at the end of the year to the county treasurer.

I have read this important law in your hearing that you may know the enormous scope of the work of the censors. For a full century our state, the Empire State of America, has had one consistent continuous advancing medical policy. Her purpose has been clear and plain—namely, that her people might have the services of a competent and a yet more competent, of an efficient and still more efficient medical profession. For a full century our state has steadily raised higher and higher her medical standard and at each step as the medical standard is raised, the scope of the work of the censor is enlarged, is widened, and the responsibility of censorship is increased.

In conclusion let me complete the answer to my question, "Why do we have censors and censorship in the profession of medicine?" Part of this answer has been made, the more important part remains to be spoken. I have tried to show you why our profession is attractive to the criminal; it remains to notice why the criminal, the fakir, the quack, gets the attention of the censors, gets censorship. In speaking to you of the medical history of our state I called your attention to blunders and mistakes which the fathers made with the homeopaths and the eclectics. These mistakes came from the head. In those contests the fathers' hearts beat true. Pride, honorable, creditable pride in our noble profession, led our fathers to the mistakes of 1857 and 1865. Pride, just, chivalrous, consecrated pride in our noble profession, —the pride which makes a man proclaim,—that in my father's family there are no bastards,—this is the reason why there has been, why there is, and why there ever will be censorship in the profession of medicine.

Young men, graduates of today, you have come into a goodly heritage, an honorable fellowship. As I look into your faces I read that which makes the future luminous with hope, splendid with ennobling ideals. The medical profession of the future will require less and less censorship.

The next toast, said the chairman, will interest everybody present because it deals with a "progressive" theme. He called upon Dr. T. H. McKee to speak for

"THE AUTOMOBILE."

When our honored toastmaster invited me to speak on the subject of the automobile, I wondered what in the world would be the use, reasoning in a childish sort of way that the audience would be divided into two classes—those who did not own a machine and would care nothing about them, and those who did own a machine and would think they knew all about them. But on further and more mature consideration, and in view of the intimate relations which you all hold towards these vehicles in your outdoor vocations, it seemed as though there might be a goodly number who would be willing to learn something *ex cathedra,* as it were, about these wonderful things which when you come to know and to be intimately associated with them, would almost tempt you to believe in the doctrine of the ancient Pantheists, and that they are endowed with a part of the soul universal, or mind, or intellect, call it what you will, capable of getting *en rapport* with your own. Indeed, unless we can accept some such theory, it becomes quite impossible to explain away many of the difficulties in understanding the lightning changes which take place in the character of certain machines, vagaries which can hardly be regarded as consistent manifestations of conduct of mere iron and steel, whose molecules are subject to welldefined physical and chemical laws. A usually well-behaved, well-trained, genteel automobile will ofttimes suddenly show a disposition to run amuck with a ferocity which can be explained on no other theory than that of obsession.

Having arrived at the conclusion that there was an excuse for talking automobile, and recovered somewhat from the confusion of mind incident to being selected for so responsible and distinguished a task, an unknown insignificant member of the motor cult, with nothing to his credit as a chauffeur, never having maimed a citizen even, while there were so many men connected with the University and this Association who have performed some really astonishing feats—such as climbing a telegraph pole with a touring car, hitting the same post in the same spot twice in the same hour of the same day with a big Stanhope—to say nothing of such every day incidents as knocking over a horse and wagon without killing the occupants—I approach the subject with fear and trembling, with about the same sensation, indeed, as thrills the novice every time he attempts to start his new machine, or the young man as he takes the hand of one of his first loves prepared again to vow eternal fidelity, a sensation of delicious interest and curiosity, not unmingled with anxiety, for as he turns on the spark, and seizes the crank, the same old question flits before his mind, like the old, old story that is ever new, "Will she?" or "Won't she?" And generally she won't.

The automobile was expected and anticipated many centuries ago, but like every great and beneficent agency for modern civilisation, it was compelled to await the development of a suitable psychological environment. Then it came to stay. Away back in the days of ancient Nineveh, Nahum, being in prophetic mood, declared:

"Chariots shall rage through the streets,"
"They shall jostle one against the other in the broad ways;"
"They shall seem like torches, they shall run like lightning."

Now, it is more than evident that Nahum did not have in mind a $5,000 four-cylinder Packard with its gentle purr when he wrote that, but a Pierce motorette; no other chariot could adequately fill the description as to raging through the streets.

Then you have to come on down the historical line for several centuries for the next reference, to about the year 1485, when was given that wonderful prophecy with which you are all more or less familiar, and credited somewhat irreverently to a lady known as Mother Shipton. In the first lines she declares: "Carriages without horses shall go, and accidents fill the world with woe."

Then, of course, we should expect to find some reference to them in those marvelous literary productions which seem to embrace almost all lines of human activity, and sure enough Will Shakespeare, in Pericles, makes Helicanus say of Antiochus:

"Even in the height and pride of all his glory, when he was seated, and his daughter with him, in a chariot of inestimable value."

If that does not refer to an automobile, in the plainest terms possible, then shall we have to call in the aid of some form of specialised higher criticism to interpret the simplest lines of the English classics. There is no other form of vehicle known to historians, ancient or modern, for which you cannot almost instantly make a fair estimate of value. Not so with the automobile, because you have absolutely no means of determining what the cost of maintenance will be for twenty-four hours, nay, even for twenty-four seconds at a time; and Shakespeare, with his inimitable art of crystallisation, in describing it as a chariot of inestimable value, makes two inferences indisputable: first, that he referred to an automobile; and, second, that he knew the nature of it. If further confirmation be required, you have but to turn to The Merry Wives of Windsor, and there you find these pregnant words:

"Take all or half for easing me of this carriage."

The honor of inventing the first modern machine undoubtedly belongs to Cugnot, of Paris, who constructed one in 1769. You will note that this was before the days of the steam engine, yet this machine ran by steam; and that it belonged to the genus automobile is conclusively established by the fact that it tore down the fence or wall around the enclosure in which it was kept. No more convincing genealogical evidence would be demanded in any court of heraldry.

The American revolution which involved the English, French and Americans,—the only people, you will note, who were inclined to invent and perfect so great a time-saving contrivance,—absorbed all their attention for the next generation, to be almost immediately followed by the Napoleonic nightmare. So it was not until 1829 that the automobile reappeared, and this time in England. Previous to this, transportation in Great Britain had been monopolised by the canals with most exorbitant and oppressive rates,—probably with rebates,—but of that history sayeth naught. As a natural result of the necessity to overcome this monoply, railroads had been constructed, and were just nicely getting under way when the automobile appeared. Their stockholders and incorporators promptly scented danger to vested interest and prospective profits in the further development of the huge machines, which were scurrying about in the early thirties between London and nearby towns, some of them making as high as 35 miles an hour,—no mean speed for a modern car,—and lost no time in endeavoring by all means in their power to arouse antagonism against this new method of locomotion. England was at the time cursed with the doctrine of state rights in an aggravated form; that is, the different counties and boroughs seemed to have absolute jurisdiction over the vehicles which were permitted to operate on their several highways. All sorts of vexatious regulations were applied, to be consummated finally by a master stroke on the part of some rural Solon who, if among us today, would undoubtedly find fame—and fortune—in the legislature of the state of New York, in an enactment which required every automobile to be preceded by a man on foot carrying a red flag. As the English have a peculiar habit of enforcing their laws, and are proverbially slow at seeing a joke, this promptly acted as an effective speed limit, and the automobile disappeared for all practical purposes for another fifty years, to reappear in France in the early seventies, where it received the impetus for its present vogue. You see, its intellectual environment was not yet ready for it.

But it is far from my intent to bore you with the history of the development of these things, so strangely human in their traits, but merely to point out that they were destined to play so high a rôle in the world's sociological evolution as to be foreseen, and in some measure anticipated, by the prophets and seers of all past ages; furthermore, that it has been reserved for you to have the high privilege of witnessing its rapid passage through those phases which mark the development of all civilising inventions, to be first the plaything of the wealthy, then the necessity of the poor. As to the part it shall play in the future, nothing short of omniscience should have the temerity to predict. Undoubtedly the greatest civilising factor of the past century, the greatest wealth producing agency, the most potent element in the diffusion of culture, and in the last analysis the most effective and eloquent peace advocate has been rapid transit; in other words, the annihilation of distance, so that the city of London is nearer to Buffalo tonight than was Detroit in the days of our grandfathers; and there are more people in this city tonight who have been immeasurably broadened and enlightened by a sojourn in distant China and Japan, than there were in those days who had visited the Mississippi river. The automobile is doing for every community locally what the railroad and the steamboat have been doing for the nations continentally.

As a healthful exercise, barring accidents, driving a machine at top speed has few equals. You who have not had experience may be mildly astonished at that statement, but I assure you that a day's driving, even about a city, will leave you at night with a ferocious appetite; while if you are nervous, irritable, tired, ready to war with all mankind, on the verge of neurasthenia, take out your car at night when you have a clear fair way, drive it for all there is in it, and you will come back—if you do come back—invigorated, rested, nerves steadied, at peace with the world, and ready for a night's sound sleep. Just think of it, you poor unfortunates, who are obliged to waste the beautiful seasons riding in street cars, what it will mean, a short time hence, to get out early on a Sunday morning, when the fruit trees are all enveloped in clouds of pink and white, and the caller air is laden with their fragrance, to see the sun rise and hear a myriad of songsters praising Almighty God for a world of delights that sluggish man can neither appreciate nor despoil, and at a good clipping gait make the run to Williamsville, back through the parks to see brown bruin get his matutinal loaf, down to the Front, and home in time for breakfast with the rest of the family, with an appetite and a disposition of so noble a character as to astonish even those who are accustomed to your performances!

But alas! the law of compensation steps in with the automobile as in all human affairs and decrees that even this great good cannot be regarded as an unmixed blessing. In fact, a severe arraignment may be made against it, on the ground that its tendencies are decidedly towards race suicide, not only individually, but collectively; in that it was never intended, nor is it in any wise adapted to the important and serious business of love-making. The trend of present social developments is all against marriage, and the explanation is exceedingly simple. A man almost never marries a human being; he falls in love with an ideal which he has contrived to build up with some fair one as a nucleus, and if he reaches the crucial point before the mists of ideality have been dissolved he is booked for life, or until freed by due process of law. But if he wait too long, that is until the ideal investments have dropped away, and he has nothing left but the mere human. there probably will be no wedding; and the oftener this occurs, the less the probability that there ever will be one for that individual. Now that is just where the automobile gets in its pernicious work. The young man who has one hand on the steering gear, the other on the throttle, one foot on the brake, and the other on the bell or reverse, with mind intently bent on nails, broken horseshoes, holes in the road, broken glass, children, dogs, and the like, with an ever present consciousness of $150 to $300 worth of rubber in jeopardy beneath him, the while trying to figure out whether the occasional missfire is due to a battery connection, a sooty plug, a trembler spring, or trouble in the carburetter, is not in an advantageous position to play the part of Romeo; and the girl in goggles and dust colored coat, with hair pointing to all the stars in the milky way, trying to carry on a conversation in a voice pitched above the roar of the machinery, with a youth so intently preoccupied as the one in question, is not a subject which can be readily idealised. And then there are incidents, incidents which are prone to occur. For instance, if a tire blow out, or a valve break, or an infinitesimal drop of water gets into that carburetter, or one of a dozen other things happens, which surely will happen in due time, and the ideal has to sit by the roadside fanning her rosy countenance with a burdock leaf, while she makes crazy suggestions about the machinery, and Apollo works and perspires, and says things audibly or inaudibly, it gets too perilously near to a realisation of the prayer of the immortal bard when he begged:

"O wad some power the giftie gie us
To see oursel's as others see us."

Under such circumstances the automobile can never be regarded in the light of a matrimonial accelerator.

Finally, gentlemen of the graduating class, as prospective experimenters in the near future with this many-sided mechanism, I cannot refrain from giving you a bit of what might not inaptly be styled comparative philosophy which was taught me by one of your professors,—whose opinions we all highly esteem,—before I ever owned or drove a machine of my own, but whose observations I have since been compelled to confirm. He would usually begin his homily with the following text: "Mac, there are no two things in heaven above or the earth beneath, so far as the evidence goes to show, that are so much alike as an automobile and a woman." And then he would proceed to elaborate something after this fashion:

> You see both are constructed with the most finely adjusted and delicately balanced mechanism of their species; both are endowed with a mind inscrutable. You study out a certain train of symptoms, and decide that they indicate a given something wrong with the machinery. You wait triumphantly for that given something with its former train of symptoms to go wrong again, for you will know just where to put your finger on the cause of the trouble; and behold the trouble comes, and the symptoms appear exactly as expected; then you get out your screwdriver and your wrenches, and put your finger on the cause of the trouble; but, like the Irishman's pulex irritans, it isn't there, it is somewhere else. And that experience you will keep repeating no matter how long you run a machine, and that, my young and unsophisticated friend, will be your experience, no matter how long you live with a woman.

Then the machine would stop, and after half to three-quarters of an hour's hard work, and we were aboard and nicely under way, he would proceed with his analogy:

> When an automobile misbehaves, you cannot force the issue; it must be induced gently to take up its burdens. If you become too hasty, or strenuous in your management, bad results, and costly, not to say dangerous will follow. You may save yourself a world of trouble if you will apply the same rule in dealing with your wife:
>
> "For where's the man who has the power or skill
> To stem the torrent of a woman's will?
> For if she will, she will, you may depend on't,
> And if she won't, she won't,
> And that's the end on't."
>
> Every spring it will require about a hundred dollars, or a little more, for new tires, a coat of paint, and a few sundries. About the same time, the lady of the house will require a new gown, an Easter bonnet, and a few sundries,—about a hundred will do it. When the automobile is possessed of a proper spirit and attending strictly to its automobile business, and not interfering with yours, it is the most efficient assistant, and the greatest comfort in life, bar one; when the presiding genius of the household is in amiable mood, attending strictly to her wifely duties, not running a caucus of suffrage clubs, she is the one, bar none.

Thus he would go on as we rode from day to day, ever eliciting new and striking parallels, and winding up with Pope's apostrophy to both:

> "Hail wayward queen
> Who rules the sex to fifty from fifteen,
> Parent of vapors and of female wit,
> Who gives the hysteric or poetic fit,

On various tempers acts in various ways,
Makes some take physic, others scribble plays,
Who cause the proud their visits to delay
And send the godly in a pet to pray!"

The witticisms of Dr. McKee's speech were received with shouts of laughter during its delivery, and generous applause was accorded the speaker at the finish.

The next toast the chairman said was of great importance to physicians and he called upon Dr. William Warren Potter to respond to

"THE PRESS"

which he did in the following words:

While sitting here listening to these very entertaining speeches, my mind carried me back thirty years,—pardon me if I grow a trifle reminiscent,—to the first public meeting of the Alumni Association, which was held almost on this very spot. Saint James's Hall, which stood on this ground, was the scene of a brilliant gathering February 23, 1875. Its capacity was 2,500 and it was filled to its limit. It was then the custom to have two addresses from the stage on commencement night. Dr. James P. White delivered the doctorate address and it fell to my lot to give the address to the association.

Indeed, it may not be inappropriate for me to state that I had something to do with organising the Alumni Association. I proposed the scheme about 1870 in a letter to my relative and friend, Prof. M. G. Potter, and later he and some others took the matter up. But it was not until 1875, that plans crystalised sufficiently to begin operations as a corporate organisation.

Do not understand that I lay claim to being the "sole inventor" of the association, or the sole responsibility for its existence in the sense that the Kentuckian claimed to have instigated a riot. Being questioned on the witness stand, he said: "I orter know something about it, I got it up."

I did not get it up in this sense, but what is of supreme satisfaction to me, in view of the importance of the association and the work it has done for thirty years, is that I did play a part, humble though it may have been, in organising and putting upon its feet this splendid association.

But I must not forget the subject you have assigned to me—"The Press." I shall speak of the medical press which is the only branch of the press of which I have any knowledge. Here, again, I must crave indulgence if I relate a bit of history. This sentiment is closely allied to the history of the Buffalo Medical Journal, and the history of the Journal is again intimately associated with that of the medical college,—the Medical Department of the University of Buffalo. Some of you will recall the fact that the Journal was established in 1845, and that the college began its career in 1846, both practically being established by the same men. For ten years the Journal was edited by that distinguished teacher of practice, Dr. Austin Flint, and the chief contributors to its columns were the faculty of the college.

In 1853, Dr. Sanford B. Hunt became the associate of Dr. Flint in the conduct of the Journal, and for the next two years was responsible for its progress. In 1855, when Dr. Flint retired, Dr. Hunt became sole owner and so continued until 1858. It was my good fortune to become Dr. Hunt's pupil in 1855, and perhaps it was through the influence of this environment that editorial work became attractive to me in later years.

In 1858, Dr. Austin Flint, Jr., assumed control, continuing for two years and then came trouble. Dr. Flint's interests were principally in the city of New York, and he put the publication of the Journal into the hands of an ambitious druggist, who soon swamped it and it disappeared from the field for a few months. It was revived by Dr. Julius F. Miner, in 1861, and continued in his hands until 1879, though edited a part of the time by Dr. Edward N. Brush.

Dr. Miner's failing health caused him to part with the Journal and it passed into the hands of a syndicate, consisting of Drs. Lothrop, Davidson, Howe, Mynter, and Van Peyma. A little later, Drs. Howe, Mynter, and Van Peyma withdrew, and Drs. Lothrop and Davidson became its owners, with Dr. Davidson as managing editor. Upon Dr. Davidson's death in 1888, his part of the Journal passed into my hands, at which time I assumed its editorial management. A few years afterward I purchased Dr. Lothrop's interest, since which time I have been sole owner, editor, and proprietor of this venerable, but always youthful representative of the medical press, now nearing the close of its sixtieth year of publication.

My object in tracing briefly this history has not been without purpose. I wish to point out the fact that for 34 years—namely, from 1845 to 1879, the interests of the college and the Journal were closely allied, each being under control of a common influence and each seeking the promotion of the other's welfare. But, in 1883, with the establishment of Niagara Medical College, came the parting of the ways, and for the next five years, the editors being professors in the new institution, the Journal practically became an organ of the Niagara college.

When I assumed the managing editorship in 1888, I began a systematic attempt to narrow the breach that had been created between the Journal and the profession, through the differences of the two medical colleges. Finally, upon the disappearance of Niagara medical school in 1898, there was no longer a formidable barrier to the accomplishment of this purpose. Nevertheless, the feeling which ran so high among the leaders and which continued for 15 years, made it a difficult task. But I have never despaired of ultimate success; and the present seems to be an appropriate occasion on which I may make an appeal, not only for a closer unity of the profession of medicine in Buffalo, but for the unanimous support of the faculty and alumni of the medical department of this great university. The Journal can make itself useful to you, and I am sure you can make yourselves useful to the Journal. Let us then strike hands in a common warfare against the inroads of disease, for the promotion of the public health, and in the interests of higher medical education! In these remarks, be it understood, I make no reflection upon Niagara University Medical College. Her numerous alumni of distinction, here and elsewhere, testify to the strength of that institution. She performed a part in elevating the standard of medical education in this state, and when her services were no longer required she gracefully retired from the field and her teachers were joined to the Buffalo University.

I must again crave indulgence for introducing a delicate topic, but it must be incorporated as an essential part of the discussion of this subject. No matter how loftily, no matter how scornfully, the soul may look down upon the filthy lucre, the hand is ever open for its reception. There are two things that must be fed to the press, or it will stop; these are, copy and money. There is no more inexorable creditor than the printer. He must have his money and have it *now,* or he will cease to set type. The printing bills of a monthly periodical must be paid every month or else the press will stop. Postage, too, is another item on the "no trust" side of the account. It costs 24 cents a year to deliver every Journal in this city,—more than it does to send it to San Francisco,—and when this is multiplied several hundred times, it shows a yearly outlay of magnitude. In spite of many perplexities, it is the pride of the Journal which I have the honor to represent, that it pays its bills when due and reaches its subscribers with regularity and promptitude. Unless these two conditions are met, no journal can survive for very long.

The other demand of the press is for copy, which is only second to its outcry for money. Original contributions must be read and corrected; discussions must be edited; society proceedings reported by the several secretaries thereof and supplied with promptitude; abstracts must be prepared; editorial material written; books must be reviewed; an aggregation of miscellaneous duties performed; and, finally, proof must be read, not once but many times,—all these and much more are necessary to make up the magazine in presentable form. And yet, almost everybody thinks he can edit and manage the magazine better than the editor.

Let me venture here, to offer a little advice. In that clever love story by Hamlin Garland, entitled, "Her Mountain Lover," the hero, Jim Matteson, in a conversation with his cultured partner, who wishes to pack him off to Europe to promote a mine in which they are interested, and who suggests that he need not fear the voyage as his wife will give him good advice which will prevent sea sickness and other discomforts, is made to say—"Advice wont hurt a feller if he don't foller it, will it?" The advice I wish to offer, especially to the recruits that have been mustered in today, is to subscribe for your home journal, pay your subscriptions promptly, send your contributions to it, and supply it with all the news items of which you become possessed. It will take an interest in you and promote your professional welfare, and in return you must take an interest in it and promote its welfare. This applies to whatever location you may select. It may not be backed by a large publishing house, hence cannot furnish hundreds of reprints in return for your contributions, but it can and will benefit you in far more important ways.

Do not feel, before you contribute to its columns, that you must discover some new disease or method of treatment; for there is nothing new under the sun. The most that is new is either in description or location, something that is known as having already existed and is given either a new name or a new place. In order to entertain the subscribers of a journal a variety of subjects must be printed during the year. Some desire articles on medicine, some on surgery, some on gynecology, and others are interested in prescriptions, hence I repeat, the entire field of medicine must be covered in order to satisfy the various wishes of readers.

Since the Buffalo Medical Journal was established, other journals have entered the field in this city and in turn have disappeared after more or less brief careers, leaving the old journal as the only oracle of the profession between Albany and Detroit on the one hand, and Toronto, Pittsburg and Cleveland on the other. It appeals to every alumnus for support, not in the spirit of mendicity, but as deserving the loyal adherence of each from its own inherent worth, and because, further, that it can and will render quid pro quo, thus making the interests reciprocal. The joy of doing should be the medical editor's ideal—his shibboleth—but a large subscription list gives practical effect to his joyful career.

According to the distinguished regius professor of medicine at Oxford, if he is correctly reported, the journal, not to say its editor, having reached the age of 60 years, should be put to its eternal rest by an euthanasia secured through a delightful anesthetic. Perhaps the editor is an appropriate candidate for that charming method of shuffling off, but not the journal, which I trust will continue on and on during many future years to be the exponent of progress in medical science. The profession of Buffalo is a stalwart body of medical practitioners, not excelled by that of any other American city of similar size. It is not difficult to believe that the medical college and the journal have contributed in a large measure to this superb condition; and may the future witness these two exponents of medicine working side by side, even hand in hand, endeavoring to solve the problems that are still to be unfolded in the realm of science!

Dr. Rochester asked the assemblage to join in singing the "Star Spangled Banner," which was done; and then he announced that the festivities were over. And so ended, amid much enthusiasm, the annual meeting of 1905.

---

The following names were registered as attending the meeting, May 3-4, 1905:

1875 J. H. Greene, Dubuque, Ia.
1859 Wm. Warren Potter, Buffalo
1891 DeWitt H. Sherman, Buffalo
1865 Henry Lapp, Clarence
1895 Sidney D. Wilgus, New York
1895 W. Grant Cooper, Ogdensburg
1885 Wm. J. Coyle, Windsor Locks, Conn.
1895 C. A. Weinbach, Buffalo
1888 A. L. Benedict, Buffalo
1893 A. T. Lytle, Buffalo
1884 DeLancey Rochester, Buffalo
1889 Grover W. Wende, Buffalo
1900 Edgar R. McGuire, Buffalo
1902 Wm. Ward Plummer, Buffalo
1866 E. L. Shurly, Detroit, Mich.
1888 E. H. Tweedy, Buffalo
1898 Charles L. Preisch, Lockport
1901 H. R. Trick, Buffalo
1885 Walden M. Ward, N. Collins
1866 M. B. Shaw, Eden
1889 Herbert U. Williams, Buffalo
1901 J. B. Frisbee, Buffalo
1875 J. P. Colgrove, Salamanca
1848 P. K. Stoddard, Prattsburg
1895 M. Axford, Buffalo
1893 Franklin W. Barrows, Buffalo
1885 Sewell A. Brooks, N. Java
1902 Charles A. Bentz, Buffalo
1895 Marshall Clinton, Buffalo
1875 H. J. Ashley, Machias
1897 L. E. Curtice, Buffalo
1874 Wm. J. Howe, Scottsville
1902 J. H. Kellogg, Bemus Point
1869 George W. Pattison, Buffalo
1871 D. W. Harrington, Buffalo
1899 Geo. S. Staniland, New York
1865 O. A. Tompkins, Randolph
1882 Eli H. Long, Buffalo
1901 Arthur Eisbein, Buffalo
1894 Cora B. Lattin, Buffalo
1903 Lawrence A. Highland, Buffalo
1895 L. C. Green, Panama
1884 W. J. French, Hamlet
1885 James F. Sherman, Rochester
1902 Edward C. Mann, Buffalo
1898 Thomas H. McKee, Buffalo
1885 F. T. Noeson, Bear Lake, Pa.
1895 W. G. Grove, Buffalo
1885 J. C. Wheeler, Buffalo
1895 M. F. Mudge, Johnson's Creek
1895 Wm. J. Austin, Perry
1895 R. Kimpton, Buffalo
1902 Otto K. Stewart, Canisteo
1902 George W. Seitz, Buffalo
1891 F. H. Van Orsdale, Belmont
1900 J. B. Young, Buffalo
1896 N. L. Burnham, Buffalo
1895 R. H. Fisher, Spencer
1891 W. H. Loughhead, Jr., Nile
1848 A. G. Ellinwood, Attica
1885 A. H. Baker, Elmira
1870 Charles Meine, Germania, Pa.
1851 Thos. D. Strong, Westfield
1875 Z. J. Lusk, Warsaw
1897 F. C. Busch, Buffalo
1901 T. M. Leonard, Buffalo
1895 G. N. Jack, Depew
1895 C. L. Randall, Franklinville
1891 Irving W. Potter, Buffalo
1872 Robert Hebenstreit, Buffalo
1894 H. C. Rooth, Buffalo
1883 Wm. H. Thornton, Buffalo
1891 Jane W. Carroll, Buffalo
1885 Frank F. Dow, Rochester
1875 Wm. P. Clothier, Buffalo
1883 B. G. Long, Buffalo
1878 B. S. Swetland, Brockton
1891 Gertrude E. Beebe, Buffalo
1880 W. R. Campbell, Niagara Falls
1892 Benj. W. Readshaw, Buffalo
1879 Benj. F. Rogers, Buffalo
1876 B. H. Putnam, North East, Pa.
1887 Eugene A. Smith, Buffalo
1887 F. H. Stanbro, Springville
1897 W. L. Phillips, Buffalo
1890 C. J. Reynolds, Buffalo
1884 George F. Cott, Buffalo
1904 Robt. F. Sheehan, Jr., Buffalo
1889 E. A. Forsyth, Buffalo
1898 F. W. Filsinger, Buffalo
1886 A. L. Richman, Morton
1883 R. W. Bamber, Waterport
1884 E. M. Shaffner, Great Valley
1887 Fridolin Thoma, Buffalo
1878 Charles G. Stockton, Buffalo
1893 A. E. Woehnert, Buffalo
1889 Allen A. Jones, Buffalo
1897 Chas. J. Rosengren, Buffalo
1899 W. W. Grantier, Buffalo
1893 J. Ullman, Buffalo
1889 Henry J. Mulford, Buffalo
1888 Henry C. Buswell, Buffalo
1888 G. H. McMichael, Buffalo
1881 C. C. Frederick, Buffalo
1866 Conrad Diehl, Buffalo
1873 Joseph Fowler, Buffalo
1897 Charles J. Mengis, Buffalo

| | |
|---|---|
| 1875 H. F. Fullerton, Buffalo | 1896 Myron M. Metz, Williamsville |
| 1904 J. A. Ragone, Niagara Falls | 1895 N. G. Russell, Buffalo |
| 1895 Chas. R. Borzilleri, Buffalo | 1895 B. H. Brady, Buffalo |
| 1889 A. W. Henckell, Rochester | 1896 Avery K. Brodie, Brooklyn |
| 1889 J. E. Widner, Chicago, Ill. | 1880 Joseph Morris Lewis, Elba |
| 1882 Sarah H. Perry, Rochester | 1883 C. E. Bowman, Alden |
| 1880 Mary J. Slaight, Rochester | 1888 Henry G. Bentz, Buffalo |
| 1895 J. Anderson, Cleveland, O. | 1889 John R. Gray, Buffalo |
| 1867 Henry R. Hopkins Buffalo | 1878 Ernest Wende, Buffalo |

The following classes held reunions:

Class of 1865—President, George M. Palmer, Warsaw. In attendance—Henry Lapp, Clarence; Orrin A. Tompkins, Randolph.

Class of 1875—President, Joel H. Greene, 481 Locust street, Dubuque, Ia. In attendance—Joel H. Greene, Dubuque, Ia.; Harmon J. Ashley, Machias; William P. Clothier, Buffalo; John P. Colgrove, Salamanca; Henry F. Fullerton, Buffalo; Zera J. Lusk, Warsaw.

Class of 1885—President, Francis S. Comfort, Campden, Ont., Can. In attendance—Asbury H. Baker, Elmira; Sewell A. Brooks, North Java; William J. Coyle, Windsor Locks, Conn.; Frank F. Dow, Rochester; Frank T. Noeson, Bear Lake, Pa.; James F. Sherman, Rochester, N. Y., Walden M. Ward, North Collins; James C. Wheeler, Buffalo.

Class of 1895—President, Sidney D. Wilgus, 47 East 58th street, New York. In attendance—Sidney D. Wilgus, New York; Montressor Axford, Buffalo; Charles R. Borzelleri, Buffalo; W. Grant Cooper, Ogdensburg; Robert H. Fisher, Spencer; LeRoy Green, Panama; W. G. Grove, Buffalo; George N. Jack, Depew; Charles L. Randall, Franklinville; N. Gorham Russell, Buffalo; Christian A. Weinbach, Buffalo.

Class of 1895 (Niagara)—President, Murray F. Mudge, Eagle Harbor. In attendance—Murray F. Mudge, Eagle Harbor; John Anderson, 405 Lincoln avenue, Cleveland. O.; William James Austin, Perry Center; Bernard H. Brady, Buffalo; Marshall Clinton, Buffalo; Richard Kimpton, Buffalo.

Class of 1855—President, Webster Wynn, Dixon, Ill. No member present.

